# A Systematic Review on Hate Speech among Children and Adolescents: Definitions, Prevalence, and Overlap with Related Phenomena

**DOI:** 10.1177/15248380221108070

**Published:** 2022-06-22

**Authors:** Julia Kansok-Dusche, Cindy Ballaschk, Norman Krause, Anke Zeißig, Lisanne Seemann-Herz, Sebastian Wachs, Ludwig Bilz

**Affiliations:** 1 Department of Health Sciences, 38871 Brandenburg University of Technology Cottbus-Senftenberg, Cottbus, Germany; 2 Department of Educational Sciences, 26583 University of Potsdam, Potsdam, Germany; 3 National Anti-Bullying Research and Resource Centre, Dublin City University, Dublin, Ireland

**Keywords:** hate speech, bullying, adolescents, children, youth, systematic review

## Abstract

Little is known about the current state of research on the involvement of young people in hate speech. Thus, this systematic review presents findings on a) the prevalence of hate speech among children and adolescents and on hate speech definitions that guide prevalence assessments for this population; and b) the theoretical and empirical overlap of hate speech with related concepts. This review was guided by the Cochrane approach. To be included, publications were required to deal with real-life experiences of hate speech, to provide empirical data on prevalence for samples aged 5 to 21 years and they had to be published in academic formats. Included publications were full-text coded using two raters (κ = .80) and their quality was assessed. The string-guided electronic search (ERIC, SocInfo, Psycinfo, Psyndex) yielded 1,850 publications. Eighteen publications based on 10 studies met the inclusion criteria and their findings were systematized. Twelve publications were of medium quality due to minor deficiencies in their theoretical or methodological foundations. All studies used samples of adolescents and none of younger children. Nine out of 10 studies applied quantitative methodologies. Eighteen publications based on 10 studies were included. Results showed that frequencies for hate speech exposure were higher than those related to victimization and perpetration. Definitions of hate speech and assessment instruments were heterogeneous. Empirical evidence for an often theorized overlap between hate speech and bullying was found. The paper concludes by presenting a definition of hate speech, including implications for practice, policy, and research.

Hate speech has become a concern in communities across the world, attested to by transnational political and civic efforts to reduce its prevalence and minimize its consequences (see initiatives by EU Commission, 2016; United Nations, 2019). The popularity of the term hate speech in public discourse is associated with a “panoply” of differing definitions (Brown, 2017, p. 422); no clear consensus has yet emerged on the essential constituents of hate speech. Children and adolescents are deemed to be especially vulnerable to the detrimental effects of this phenomenon. Recent research has revealed that young people who are exposed to online hate speech (cyberhate) commonly experience negative feelings (e.g., anger, sadness or shame; UK Safer Internet Centre, 2016) as well as diminished levels of trust (Näsi et al., 2015). Exposure to hate speech may also be associated with processes of political radicalization (Bilewicz & Soral, 2020). Victims may lack appropriate coping strategies to mitigate the harm of hate speech (Krause et al., 2021) and even seek revenge (Wachs et al., 2020). Therefore, it seems crucial to carefully monitor how frequently children and adolescents encounter hate speech.

One is faced with several issues when one attempts to access research on the prevalence of hate speech among young people. Firstly, evidence suggests that definitional ambiguities of hate speech are prevalent in academic research (as shown for Germany by Sponholz, 2020). Without a precise theoretical definition of hate speech and the ability to soundly differentiate it from related phenomena, empirical research may lack validity. For research on children and adolescents in particular, hate speech needs to be differentiated from bullying (e.g., Lehmann, 2019). Globally, more than 30% of young people report that they have been bullied by their peers (United Nations Children Fund, 2018). Thus, a theoretical and empirical need arises to clarify whether or not hate speech and bullying have identical features and to what extent they are jointly experienced by young people. Secondly, there is also an issue with the comparability of frequency measures. Lehmann (2019), for instance, reports that, within a US-based sample of 12-to-18-year-olds, 26% were exposed to hateful language at school, whereas Wachs et al., 2019 reports a rate of 36.2% for US-based students of a comparable age. These differences must be contextualized in terms of definitions and assessments. Finally, a recent narrative review (Bauman et al., 2020) on the frequency of online hate speech (cyberhate) among children and adolescents acknowledges a variety of definitions and assessment methods and a paucity of empirical studies focused on young people. This review, however, contains little information regarding hate speech in offline contexts. Hence, the state of the literature calls for a systematic examination of empirical studies on the prevalence of hate speech among children and adolescents. In this sense, a focus on online and offline modes is important. Because, despite the rising developmental significance of the internet (specifically social media) as a socialization context for adolescents (Boer et al., 2020), hate speech incidents may still also take place in the context of face-to-face encounters (e.g., with peers or teachers at school; Krause et al., 2021). There may be links between on- and offline experiences of hate speech. In summary, there is a need for a systematic examination of existing research on hate speech frequencies (online, offline) in children and adolescents. This review will contribute to the ongoing academic debate on hate speech definitions and assessment methods, and clarify how hate speech potentially overlaps with related phenomena. It will also provide an empirical basis for measures aimed at preventing and mitigating the harm of hate speech among young people.

## Research Questions

The following research questions (RQ) are derived from this gap in the literature:

(1) How does research on the prevalence of hate speech among children and adolescents define hate speech? How prevalent is hate speech among children and adolescents (age 5 to 21 years), and do group differences based on demographic features exist?
(2) How does research on the prevalence of hate speech among children and adolescents differentiate between hate speech and other forms of violence (e.g., bullying)? Is there evidence for an empirical overlap of hate speech with other forms of violence?

## Methods

We used the Cochrane Collaboration Handbook for Systematic Reviews (Higgins et al., 2021) to structure the review methodology. The prevalence or frequency of hate speech was framed with the bystander-perpetrator-victim paradigm (for a review, see Vollhardt & Bilewicz, 2013). The paradigm is sensitive to the social dynamics and roles that are often concomitant with acts of interpersonal violence. It has been popular in empirical research on human conflict and victimhood (e.g., for bullying; Zych et al., 2015).

### Search Strategy

The search-string was composed using the following terms: [hate speech OR offensive speech OR offensive word* OR hate-related speech OR hate-related word* OR hateful speech OR hateful words* OR cyber-hat* OR cyberhat* OR online hat* OR digiti?ed hat*] AND [child* OR youth OR adolesc* OR adoles?en* OR young adult* OR pupil* OR student*].

Because we were interested in academic research conducted internationally as well as in Germany—where this systematic review supports a forthcoming field-survey—the search string also considered German equivalents of “hate speech.” We searched a number of academic databases, including Psyndex, Psycinfo, SocIndex, and ERIC on August 7, 2020. No limit was set on publication dates. Discussions with the authors’ professional network also led to a number of relevant publications that were not in the database and this research was inputted manually.

### Inclusion and Exclusion Criteria

Citations and abstracts of the publications retrieved were exported to a reference management system. Duplicates were removed using automatic and manual detection. The first author screened titles and abstracts and removed records that obviously failed the inclusion criteria. Two raters then independently checked inclusion and exclusion criteria of this reduced pool of publications based on their full text. This second step created the final pool of publications.

Publications were included in the review if they fulfilled four specific criteria. Conceptually, the publication had to deal with real-life experiences of hate speech (online and offline), meaning that it had to include people involved in hate speech as a perpetrator, a victim, or a witness (criterion one). Publications from the areas of computer science or mathematics, which report frequencies established by algorithmic hate speech detection (e.g., in social media tools) were out of scope. The publication had to report empirical results for the prevalence of hate speech (criterion two). And it had to report results for children and adolescents aged 5 to 21 years (criterion three). This age-based criterion captured young people from the point of school entry up until their late adolescence or young adulthood, which, in a number of countries, coincides with the age of legal majority. We considered research (in English or German) published in reviewed academic journals, monographs, PhD theses, and full study reports that had been made available for academic conferences (criteria four). This criteria narrows down our review to a systematization of academic research for three reasons. Firstly, this review is intended to clarify the variation of academic definitions of hate speech. Secondly, it would be extremely complicated, if not impossible, to track down all internationally available non-academic studies on hate speech, because of linguistic differences and issues with availability in academic literature databases. Thirdly, it is likely that political and civic reports will be influenced by specific interests. Academics, on the other hand, are bound to follow the principle of a conditional objectivism (Reber & Bullot, 2019). This should reduce bias in their definition of hate speech, data collection, analysis, and interpretation. Publications were excluded if they failed to meet any of these four criteria. The interrater-reliability (Cohen’s Kappa) for the down-selection was *κ* = .63, which is still considered substantial (Landis & Koch, 1977).

### Quality Appraisal of Included Publications

The validity and reliability of a publications’ results (e.g., hate speech frequencies) were assessed through a quality appraisal of each publication under consideration for this review. The overall quality could be high (H), moderate (M), low (L), or very low (VL). The quality score represents the forced-choice consensus of the two full-text coders. They first rated the quality in a double-blind procedure and then discussed their evaluation. Quality criteria for publications with quantitative results were based on the GRADE System (Ryan & Hill, 2016) and its application by Fischer et al. (2020). The basic quality evaluation of a publication was either high (hate speech or its synonyms were clearly defined, the assessment of hate speech frequencies was adequate to the definition) or low. Methodological issues triggered a downgrading; the quality was reduced if samples were insufficiently described (e.g., missing information on size, age, gender), if samples were biased (no random sampling or no information on sampling approach at all), or if they were too small (samples below 300; see Ryan and Hill, 2016). Qualitative publications were assessed with criteria developed by the first author based on Mayring (2016). A high quality was assigned if the sampling strategy was reasoned and transparent, if triangulation was applied and if the researcher’s position and interpretations were sufficiently outlined and documented.

### Coding System

The research questions and the quality appraisal guided the structure and criteria of the coding system (see Appendix 1). Two raters conducted a blinded full-text coding of the final publication sample. The calculation of Cohen’s Kappa was based on quantitative criteria only. The remaining criteria (asterisked Appendix 1) were only checked for content. Discrepancies and indecisions could be resolved through consensus-heading discussions between the two reviewers. The interrater reliability of the full-text coding system was *κ* = .80 (minimum: .11 [Empirical analysis of conceptual overlap], maximum: 1.00 [research approach].

## Results

**Figure 1.**
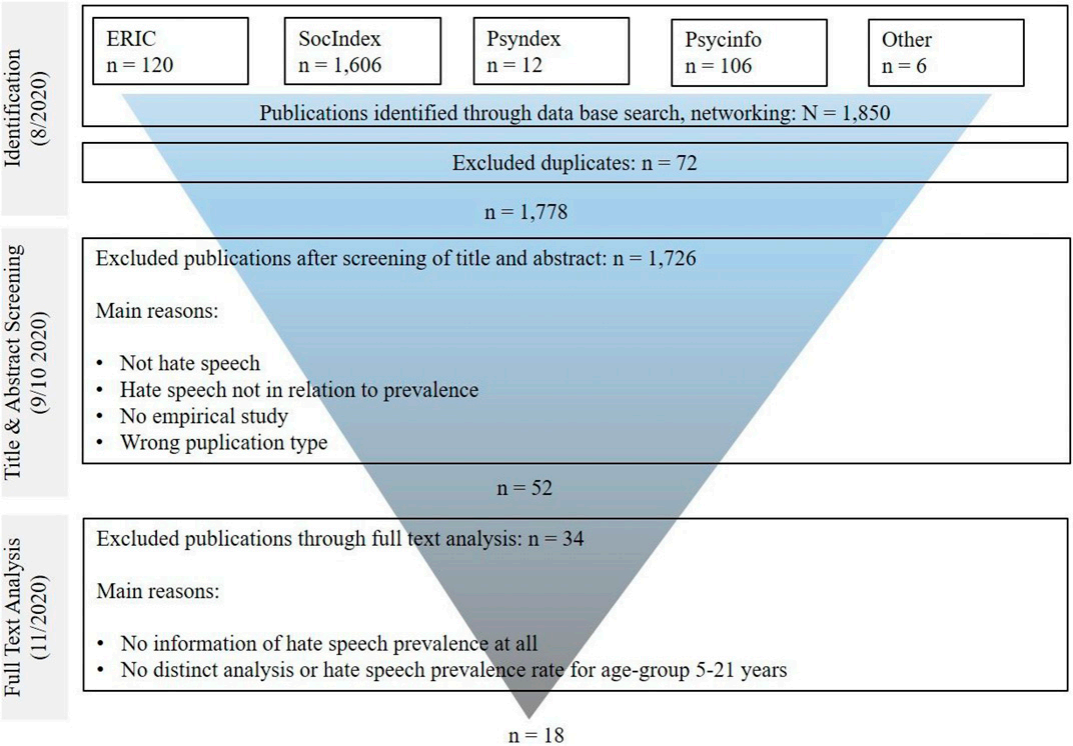
Literature search - process and Results.

In total, 1,850 publications were identified. After 72 duplicates had been removed, the title and abstract screening of 1,778 publications, done by the first author, eliminated the majority of publications - 1,726 in total. Two raters then applied the double-rating procedure (see “Search Strategy”) to the remaining 52 publications. This step produced the final pool of 18 publications (see Figure 1). Eight publications were excluded because they reported hate speech frequencies for mixed samples of adolescents and adults only (Costello et al., 2016; 2017; 2020; Costello & Hawdon, 2018; Hawdon et al., 2017; Murray, 2011; Näsi et al., 2015; Savimäki et al., 2020). Publications from the project Global Kids Online, issued by the United Nations Children Fund (e.g., Livingstone et al., 2019) were excluded due to not meeting criterion four.

### Main Characteristics of Publications Included and Underlying Studies

The 18 publications forming the corpus of this review were published between 2004 and 2021. One publication was initially included as preprint. The majority had been published in academic journals. The quality assessment of these publications revealed that four publications were of high quality, 12 were of medium quality, and two were of low quality (see Table 2 for details). The publications are based on 10 distinct studies. The majority of the studies (8 out of 10) used a quantitative methodology. Vaught (2012) applied the qualitative method of ethnography. The first research project by the cumulative PhD thesis by Tynes (2005) considered quantitative and qualitative elements. Five peer-reviewed publications referred to data from studies funded by non-academic authors (Elpus & Carter, 2016; Lehmann, 2019, 2020; Soral et al., 2018; van Dorn, 2004). Though corporate reports by non-academic authors were out of scope for this review (see section on general design principles), academic publications were included that were based on their data. Four publications (Elpus & Carter, 2016; Lehmann, 2019; 2020; van Dorn, 2004) referred to three distinguishable studies within the US National Crime Victimization Survey (NCVSS; e.g., United States Census Bureau, 2021). The NCVSS is a bi-annually conducted survey series. It assesses victimization and security risks of 12-to-18-year-old adolescents and is sponsored by the US Ministry of Education. The publication by Soral et al., 2018 referred to data from the Polish National Opinion Poll on Hate Speech, which was funded by a non-governmental organization (Stefan Bartory Foundation, 2014). Most studies collected data in North America and Europe. The only multi-country study also included countries in Asia. The samples generally consisted of adolescents up to 18 years of age. Only two studies, which refer to three publications (Blaya & Audrin, 2019; Blaya et al., 2020; Vaught, 2012), used a sample of a broader age range (8 years), spanning from early to late adolescence. No study looked at children younger than 12 years old. Subsequently this text will simply refer to adolescents, juveniles, and young people. Table 1 gives an overview of the main features of all publications and studies that were included in this review.

**Table 1.** Main Characteristics of the Research Included in this Review.

Studies (*n* = 10)			Publications (*n* = 18)		
—	n	%	—	n	%
Country of data-collection			Year		
US	5	50	2004–2009	2	11
Germany^1^	1	10	2010–2015	2	11
Finland	1	10	2016–8/2020	13	72
France	1	10	2021^3^	1	6
Poland	1	10			
Multi-country^2^	1	10			
Research approach			Type of publication		
Quantitative	8	80	Journal article	16	89
Qualitative	1	10	Dissertation	1	5.5
Quantitative & qualitative elements	1	10	Book series	1	5.5
Sample age (range)			Quality^4^		
12–18 years	5	50	High	4	22
12–20 years	1	10	Moderate	12	67
13–17 years	1	10	Low	2	11
15–18 years	2	20	Very low	0	0
13–21 years	1	10			
Sample size					
< 1000	5	50			
1001–2000	2	20			
2001–4000	1	10			
4001–6829	2	20			
Sample access					
Educational institutions	7	70			
Other (e.g. social media, internet)	3	30			
Based on study of non-academic actor					
No	6	60			
Yes	4	40			

^1^Also part of multi-country study.

^2^Cyprus, Germany, Greece, India, South Korea, Spain, Thailand, USA.

^3^Initially included in preprint status.

^4^Based on the whole publication. Different sets of quality criteria were used for quantitative and qualitative designs.

### Definitions and Prevalence of Hate Speech in Empirical Research on Children and Adolescents

To answer the first research question on definitions of hate speech, a text-based detection approach was applied to identify general terms and descriptive features of hate speech definitions (see Appendix 2). This approach was followed for 15 publications for which the full-text coding process revealed that definitions existed. Three publications (Elpus & Carter, 2016; Soral et al., 2018; Tynes, 2005) were not assessed due to missing definitions. This process created a typology of definitions that referred to the total number of descriptive features required to decide on whether a given definition was broad, medium, or narrow in scope. Hate speech frequencies were contextualized with information on assessment instruments, the sample (e.g., age), and the results of the quality appraisal. The frequencies were systematized by study and by the assessed perspective (exposure, victimization, and perpetration), and group differences were briefly outlined. Table 2 shows hate speech definitions, contextual information, and prevalences throughout all publications and studies included.

**Table 2.** Hate Speech—Definitions, Assessment, and Prevalence.

	Definition		Assessment						Sample				Prevalence Rates in %^1^		
Publications (by study)/quality^a^	Term applied	Scope	Modus	Type	Items based on	Structure, No. of items	Items reflective of HS-features	Reference period	*N*	Age	Recruit-ment	Country	Exposure	Victim-ization	Perpe-tration
Blaya & Audrin (2019)/M Blaya et al. (2020)/H	Cyberhate	Medium Narrow	Online	Self	Hawdon et al. (2015)	Multiple 6	Forms Modus Negative effect	6 months	1,900 1,889	12–20	School	France	43.3 —	10 9.6 to 11.4^8^	5.3 5.1
Elpus & Carter (2016)/L	Hate speech	Not given	Offline	Self	NCVSS	Single, 2	Target Forms Locations Neg. effect	12 months	26,419^3^	12–18	Public census	US	—	9	— —
Lehmann (2019)/M Lehmann (2020)/M	Hateful written words & graffiti/hate	Broad	Offline	Self	NCVSS	Single, 2	Target Forms Locations Neg. effect	12 months	4,607^3^	12–18	12–18	US	26 26	7 7	—
van Dorn (2004)/M	Hate speech/hate-related words/symbols	Broad	Offline	Self	NCVSS	Single, 2	Target Forms Locations Neg. effect	12 months	3,991^3^	12–18	12–18	US	39.2	11.9	—
Oksanen et al. (2014)/M Räsänen et al. (2016)/M	Hate material/hate speech	Medium	Online	Self	Not given	Multiple >20	Target Modus Locations Neg. effect	3 months	723 623^4^	15–18	Facebook	Finland	67^5^ —	21 23.4	10 6.7
Soral et al., 2018/M^2^	Hate speech	Not given	Not specified	Self	Own devt	Multiple 8	Target Neg. effects	Lifetime	682	16–18	Facebook	Poland	*M/SD:* 3.98^6^/1.95	—	—
Tynes (2005)/L^2^	Derogatory racial utterances/hate speech	Not given	Online	Observation	Own devt	Not given	Target (race only)	38 chat-sequences of 30 minutes	not given	13–17	Teen chat rooms	US	47^7^	—	—
Vaught (2012)/M	Racist hate speech	Medium	Not specified	Self & observation	Not given	Ethnographic protocol	Not given	6 months	Not given	13–21	Prison school	US	—	—	—
Wachs & Wright (2018)/M Wachs & right (2019)/H Wachs & Wright (2020)/H Wachs et al., 2019/H Wachs et al. (2020)/M	Online hate Online hate Online hate Cyberhate Cyberhate	Broad Broad Medium Broad Medium	Online	Self	Hawdon et al., 2017 Own devt	Single, 3 Single, 1	Target Forms Neg. effect	12 months Lifetime	1,480	12–17	School	Germany	53.7 — — — —	16.9 16.9 16.9 16.9 17.2	11.3 11.3 11.3 11.3 —
Wachs et al., 2019/M Wachs et al., 2021a/M	Online hate Cyberhate/hate speech	Medium Narrow	Online	Self	Hawdon et al. (2017)	Multiple 12	Target Forms Modus Neg. effect	12 months	6,829 (221 to 1480)	12–18	School	Cyprus Germany Greece India S. Korea Spain Thailand US Total	35.7 53.7 54 31.4 39.3 68.5 65.0 36.2 49.1	— — — — — — — — 10.7 to 18.4^9^	4.6 11.3 11.1 15.3 4.2 7.8 32.2 24.5 14.2

*Notes.* If no information is reported for a feature, then the information given for the first publication within the same study applies.

^a^Quality is coded as: H: High, M: Medium, L: Low.

^1^Ranges were reported if publications provided them (e.g., based on forms, targets or locations of hate speech).

^2^Quality appraisal was only based on one study cited in the publication (study 3 in Soral et al., 2018; study 1 in Tynes, 2005).

^3^Based on data from NCVSS (Elpus & Carter, 2016: pooled data from 2005, 2007, 2009, 2011, 2013; Lehmann, 2019, 2020: data point 2013; van Dorn, 2004: data point 1999).

^4^Sample solely based on adolescents who were exposed to hate speech (not including adolescents who lacked exposure).

^5^Oksanen et al. (2014) also report target-specific prevalences (e.g., 68% sexual orientation, 68% physical appearance, 50% ethnicity or nationality; 43% religious belief).

^6^Means (M) and standard deviation (SD) is reported as percentages were unavailable. Text stated that juveniles were “often” exposed to hate speech (scale: 1 = never, 2–3 = sometimes, 4–5 = often, 6–7 = very often).

^7^Prevalence refers to frequency of having detected at least one racial utterance within 38 conversation transcripts.

^8^Range is based on minimum and maximum related to locations of victimization (e.g., 11.4% cellphone, 9.6% social media)

^9^Range is based on minimum and maximum related to target specific forms of victimization (e.g., 10.7% online exclusion/18.4% making jokes based on religious affiliation).

#### General and Descriptive Terms of Hate Speech Definitions

A majority of publications (10 out of 15) included definitions for the terms online hate or cyber hate/hatred (e.g., Blaya & Audrin, 2019; Blaya et al., 2020; Räsänen et al., 2016; Wachs et al., 2019b, 2019a, 2020, 2021a, Wachs & Wright, 2018, 2019, 2020; see Table 2 and Appendix 2). Some definitions referred to hate speech either as a synonym of online hate or cyberhate (e.g., Oksanen et al., 2014; Räsänen et al., 2016; van Dorn, 2004; Wachs et al., 2021a), or as an exclusive term (Vaught, 2012). Broad scope definitions of up to three descriptive terms (Lehmann, 2019, 2020; van Dorn, 2004; Wachs et al., 2019; Wachs & Wright, 2018, 2019) were slightly less frequent than medium scope definitions with four to five descriptive terms (Blaya & Audrin, 2019; Oksanen et al., 2014; Räsänen et al., 2016; Vaught, 2012; Wachs et al., 2019, 2020; Wachs & Wright, 2020). Narrow definitions with six or more descriptive terms were apparent in only two publications (Blaya et al., 2020; Wachs et al., 2021a). Looking at general terms (see Appendix 2), most definitions were similar in framing hate speech either as a behavior or an action (e.g., Oksanen et al., 2014; Wachs et al., 2020; Wachs & Wright, 2018, 2020), or more specifically as communication or expression (Blaya et al., 2020; Blaya & Audrin, 2019; Räsänen et al., 2016; van Dorn, 2004; Wachs et al., 2019, 2021a). A few definitions consisted of general terms that framed hate speech as an aggressive or violent interaction (e.g., Lehmann, 2019; Wachs et al., 2019; Wachs & Wright, 2018, 2019). Lehmann (2020) was unique in framing hateful written words or graffiti as an example of incivility or group dynamics. Vaught (2012, p. 243) also stood out by defining hate speech as “messages of structural, bodily, and/or psychological subordination that are grounded in historical and contemporary ideological and structural practices of violent racial power.” In line with critical race theorist Mary Matsuda et al. (1993), Vaught connected hate speech to a shared norm that justifies any conduct aimed at upholding the power and supreme status of the dominant social group (here: white people). In this point, Vaught resembles Lehmann (2019, 2020) who defined hate-related words, graffiti, or symbols through processes of stigmatization and in- and out-group formation. Nine definitions closely (e.g., via brackets) linked their general terms to specific forms of hate speech (e.g., messages, memes, videos, or graffiti) (Blaya et al., 2020; Blaya & Audrin, 2019; Oksanen et al., 2014; van Dorn, 2004; Vaught, 2012; Wachs et al., 2019, 2020, 2021a; Wachs & Wright, 2020).

Looking at descriptive features (see Appendix 2), all definitions described a target of hate speech. Most definitions provided examples of target groups, such as nationality, ethnic background, religion, or sexual orientation. Only Blaya & Audrin (2019) and Blaya et al. (2020) narrowed down the group features exclusively to ethnic or religious backgrounds. Fourteen definitions (exception: van Dorn, 2004) stated that hate speech is necessarily directed at groups rather than individuals. Four definitions referred to out-groups (Blaya & Audrin, 2019; Lehmann, 2019; 2020; Räsänen et al., 2016). Persons or individuals were considered to be the targets of hate speech in 11 definitions (exceptions: Lehmann, 2019, 2020; Räsänen et al., 2016; Wachs et al., 2019). The second dominant feature appearing across the 12 definitions assessed were effects in terms of the harmfulness of hate speech (exceptions: van Dorn, 2004; Wachs & Wright, 2019), generally defined as the individual being belittled, defamed, humiliated, denigrated, dehumanized, threatened, or debased. Only a minority of definitions (Blaya et al., 2020; Blaya & Audrin, 2019; Räsänen et al., 2016; Wachs et al., 2021a) considered the harmfulness of hate speech in terms of a given community or social group (e.g., disintegration of social cohesion, advocacy of hatred, rejection, violence, or hostility). The idea that the perpetration of hate speech could have positive effects such as benefitting one’s in-group (by attracting new members, through in-out-grouping dynamics, building of group identities, or enforcing vertical group relations) was prevalent in only four definitions (Blaya & Audrin, 2019; Lehmann, 2020; Vaught, 2012; Wachs et al., 2021a). Aspects of perpetration that refer to a person-centered basis of hate speech behavior—such as prejudice, bias, a devaluating attitude, hatred, or, most often, the purpose, aim or intention to harm—were mentioned in nine definitions (Blaya et al., 2020; Blaya & Audrin, 2019; Oksanen et al., 2014; Räsänen et al., 2016; Vaught, 2012; Wachs et al., 2019, 2020, 2021a). Only a few definitions considered whether hate speech had occurred publicly or in private (Blaya et al., 2020), directly or indirectly (Wachs et al., 2019, 2021a).

### Assessment of Hate Speech Prevalences

To provide a context for the hate speech frequencies identified, we provide here information on assessment methods. Self-assessments were the dominant tool used to assess whether and to what extent young people experienced hate speech (Table 2). Only two publications reported observational measures (Vaught, 2012; Tynes, 2005). Single or multiple items were common and no publication hinted at a specific scale to assess the prevalence of hate speech. Items with either binary or continuous response ratings were used. Five publications, related to three studies, reported items with reference periods of 6 months or less (e.g., Blaya & Audrin, 2019; Blaya et al., 2020; Oksanen et al., 2014; Räsänen et al., 2016; Tynes, 2005). Three publications, related to two studies, used vignettes with participants being asked to consider incidents of hate speech experience over the course of their lives (Soral et al., 2018; Wachs et al., 2020). Otherwise, global items were used, generally with a reference period of 12 months (Elpus & Carter, 2016; Lehmann, 2019, 2020; van Dorn, 2004; Wachs et al., 2019b, 2019a, 2020, 2021a, Wachs & Wright, 2018, 2019, 2020). The items of Soral et al., 2018 were unique in that they consisted of specific statements that were classified as hate speech by minority group members through a pretest procedure.

The ways of assessing targets of hate speech differed across items. Blaya et al. (2020), for instance, sought responses about groups and individuals being targeted in general, whereas Wachs & Wright (2018, 2019, 2020) mentioned specific groups (e.g., religious, ethnic) and individuals. The variety of groups reflected in the analyzed items differed from a focus on two groups or less (e.g., Muslims and refugees in Soral et al., 2018; ethnic and religious groups in Wachs et al., 2019, 2021a; race in Tynes, 2005) to considering more than two groups (e.g., based on gender, religion, race, sexual orientation in Wachs et al., 2019, 2020, Wachs & Wright, 2018, 2019, 2020). The specific target group was not identified in items used by Blaya et al. (2020) and Blaya & Audrin (2019). Oksanen et al. (2014) and Räsänen et al. (2016) stood out as their items addressed more target groups than other studies. In addition to sexual orientation, ethnicity and nationality, gender, and religious beliefs, they also included disability, misanthropy, and political views.

Six quantitative studies, related to 13 publications, used items that reflected specific verbal and nonverbal forms of hate speech, such as messages, comments, words, graffiti, memes etc. (exceptions: Oksanen et al., 2014; Räsänen et al., 2016; Soral et al., 2018; Tynes, 2005; Vaught, 2012). School-based locations of hate speech (classrooms, bathrooms, hallways, areas outside of school building) were reflected only in NCVSS-based items (Elpus & Carter, 2016; Lehmann, 2019; 2020; van Dorn, 2004). Locations mediated by ICT (e.g., sites on the Internet) were referred to either globally (e.g., Wachs et al., 2021a) or with items pointing to specific Internet sites, social media tools, or technologies (e.g., Blaya & Audrin, 2019; Blaya et al., 2020; Oksanen et al., 2014; Räsänen et al., 2016). Only two studies, related to four publications, assessed whether online hate posts were searched for deliberately or not (Blaya & Audrin, 2019; Blaya et al., 2020; Oksanen et al., 2014; Räsänen et al., 2016). Cyberhate was the most commonly researched type of hate speech in this sample of publications and studies, thus the respective items logically reflected the online modus of hate speech. In two publications (Soral et al., 2018; Vaught, 2012) the modus was not specified. The NCVSS-based research (Elpus & Carter, 2016; Lehmann, 2019; 2020; van Dorn, 2004) assessed the prevalence of hate speech with two items. The first item captured offline exposure (“seeing hate-related symbols and graffiti in school locations,” e.g., Lehmann, 2020, p. 177). The extent to which the students who saw these symbols and graffiti felt victimized remained unclear. The second item (“being called hate-related names or words at school,” ibd.) likely referred to offline victimization.

### Prevalence of Hate Speech among Children and Adolescents

The systematic categorization of hate speech frequencies (see Table 2) shows which modes (online, offline) and perspectives (exposure, perpetration, victimization) hate speech frequencies were reported for, including studies and publications in this review and whether they provided specific prevalence rates (e.g., with regard to forms, targets etc.). The provision of hate speech frequencies by gender, age, or socio-economic status clarifies, as mandated by research question one, whether group differences that are based on socio-demographic criteria were identified in the included research.

The prevalence of hate speech was generally studied and reported in online contexts (Blaya et al., 2020; Blaya & Audrin, 2019; Oksanen et al., 2014; Räsänen et al., 2016; Tynes, 2005; Wachs et al., 2019b, 2019a, 2020, 2021a, Wachs & Wright, 2018, 2019, 2020). The prevalence of hate speech in offline contexts was either rarely researched and reported (Elpus & Carter, 2016; Lehmann, 2019; 2020; van Dorn, 2004) or it was unclear exactly what the context of the hate speech was Soral et al., 2018); Vaught, (2012). In terms of perspectives covered, this review found only two publications, based on two studies, that reported frequencies for exposure, victimization, and perpetration together (Blaya & Audrin, 2019; Wachs & Wright, 2018). In the remaining publications there was less coverage on adolescent involvement in hate speech. Prevalences based on specific hate-speech characteristics were reported only in six publications, namely: forms (Wachs et al., 2021a); locations (Blaya & Audrin, 2019; Oksanen et al., 2014; Tynes, 2005); targets (Elpus & Carter, 2016; Oksanen et al., 2014; Räsänen et al., 2016; Tynes, 2005); and perpetrators of hate material (Blaya & Audrin, 2019). And when specific prevalences were reported, it was only done so as it related to exposure (Oksanen et al., 2014; Tynes, 2005; Wachs et al., 2019) or victimization (Blaya & Audrin, 2019; Elpus & Carter, 2016; Oksanen et al., 2014; Räsänen et al., 2016; Wachs et al., 2021a), and only rarely perpetration (Blaya & Audrin, 2019).

Overall, frequencies for hate speech exposure were higher than those related to victimization and perpetration. Eight out of the nine quantitative studies assessed how frequently adolescents were exposed to hate speech (online or offline). Regarding online hate speech, a minimum of 31.4% of adolescents in India and a maximum of 68.5% in Spain reported that they were exposed to cyberhate or that they witnessed it (Wachs et al., 2021a). The remaining publications reported exposure rates within this range (Blaya et al., 2020; Blaya & Audrin, 2019; Lehmann, 2019, 2020; Oksanen et al., 2014; Räsänen et al., 2016; Tynes, 2005; van Dorn, 2004; Wachs et al., 2019; Wachs & Wright, 2018). For offline hate speech, exposure data were sparse. In representative samples of adolescents in the US surveyed within the context of the NCVSS, a total of 39.2% observed hate-related graffiti and symbols at school in 1999, with the frequency dropping to 26% by 2013 (Lehmann, 2019; 2020; van Dorn, 2004). It was reported that Polish Facebook users were often exposed to on- and offline hate speech (Soral et al., 2018).

Seven studies, related to 14 publications, reported quantitative data for hate speech victimization (Blaya et al., 2020; Blaya & Audrin, 2019; Elpus & Carter, 2016; Lehmann, 2019, 2020; Oksanen et al., 2014; Räsänen et al., 2016; van Dorn, 2004; Wachs et al., 2019, 2020, 2021a, Wachs & Wright, 2018, 2019, 2020). Overall victimization rates varied between 7% in a representative sample of 12-to-18-year-old adolescents in the US (Lehmann, 2019, 2020) to 23.4% in a convenience sample of Finnish Facebook users (Räsänen et al., 2016). Generally, frequencies for offline victimization (7% to 11%; Elpus & Carter, 2016; Lehmann, 2019, 2020; van Dorn, 2004) were lower than those for online victimization (9% to 23.4%; Blaya et al., 2020; Blaya & Audrin, 2019; Oksanen et al., 2014; Räsänen et al., 2016; Wachs et al., 2020, 2021a, Wachs & Wright, 2018, 2020).

Four studies, related to ten publications, reported frequencies of online hate speech perpetration (Blaya et al., 2020; Blaya & Audrin, 2019; Oksanen et al., 2014; Räsänen et al., 2016; Wachs et al., 2019b, 2019a, 2021a, Wachs & Wright, 2018, 2019, 2020). The prevalence of online hate speech perpetration ranged from a minimum of 4.2% for a population of 12-to-17-year-old adolescents in South Korea to a maximum of 32.2% for a comparable population in Thailand (see Wachs et al., 2019). In Western European countries, perpetration rates varied from 5.2% in France (Blaya & Audrin, 2019; Blaya et al., 2020) to 11.3% in Germany (Wachs et al., 2019a, 2019, Wachs & Wright, 2018, 2019, 2020). Using ethnographic methods, Vaught (2012) observed that teachers in a U.S. prison regularly perpetrated hate speech against black youth over a period of 6 months.

The main group difference reported in terms of personal characteristics was gender (Blaya & Audrin, 2019; Elpus & Carter, 2016; Räsänen et al., 2016; Wachs et al., 2019a, 2019, 2020, Wachs & Wright, 2018, 2019). Three publications provided group differences based on age (Räsänen et al., 2016; Wachs et al., 2019, 2021a) and two, respectively, on socio-economic status (Oksanen et al., 2014; Wachs & Wright, 2019) and with regards to respondents’ country of residence (Wachs et al., 2019, 2021a). Results suggest that increasing juvenile age (from 12 to 20 years) might be linked to higher exposure to online hate speech (Wachs et al., 2019). But the frequency with which people were victimized by online hate speech did not increase with age (Räsänen et al., 2016; Wachs et al., 2021a). The data identified on perpetration also indicated that males perpetrated cyberhate more frequently than females (Blaya & Audrin, 2019; Wachs et al., 2019a, 2019, Wachs & Wright, 2018, 2019), but that both groups were equally likely to become victims of cyberhate (Elpus & Carter, 2016; Räsänen et al., 2016; Wachs et al.,2020; Wachs & Wright, 2018). Socio-economic status was not associated with exposure to online hate speech (Oksanen et al., 2014), although increased affluence in the family did lead to a decrease in being victimized (Wachs & Wright, 2019).

### Overlap Issues in Empirical Research on Hate Speech Prevalence among Children and Adolescents

With regard to the second research question, we initially give a systematized account of how included publications differentiate theoretically between hate speech and other forms of violence, and go on to offer a summary of the evidence identified for there being an empirical overlap.

### Theoretical Overlap of Hate Speech and Related Terms

In 12 publications bullying was the concept that was seen to most closely overlap with hate speech and online or cyberhate (Blaya et al., 2020; Blaya & Audrin, 2019; Elpus & Carter, 2016; Lehmann, 2019, 2020; Oksanen et al., 2014; Räsänen et al., 2016; Wachs et al., 2019a, 2019, 2020, Wachs & Wright, 2018, 2019). Two of these publications mentioned bullying without providing a detailed explanation of that overlap (Elpus & Carter, 2016; Oksanen et al., 2014). The remaining 10 publications described differences and/or similarities between hate speech and bullying. Lehmann (2019), for instance, links both concepts by stating that, phenomenologically speaking, both behaviors concerned target individuals. Other authors connect hate speech and bullying via the perpetrator’s intention to harm a person or group (e.g., Blaya et al., 2020; Blaya & Audrin, 2019; Lehmann, 2020; Räsänen et al., 2016; Wachs et al., 2019, 2020; Wachs & Wright, 2019). When it comes to targeting, cyberhate and bullying both “involve individuals or communities that are chosen on specific identified or supposed characteristics” (Blaya et al., 2020, p.2). For those perpetrators identified, such characteristics included, for instance, prejudicial views of minorities (Blaya et al., 2020; Wachs et al., 2020) or toxic online disinhibition (Wachs & Wright, 2018). Looking at relationship features, power imbalances may also link cyberbullying and cyberhate (Blaya et al., 2020). The means and contexts in which both phenomena become apparent were also regarded as being similar (Blaya & Audrin, 2019; Blaya et al., 2020). Thus, both hate speech and bullying may be mediated by information and communication technologies or not (similar context or modus) and they may operate through words, pictures, or comments (similar means or forms).

The differentiators between hate speech and bullying were usually identified in the ways in which perpetrators thought about the social groups they were targeting. The analyzed literature tended to emphasize the fact that cyberhate was necessarily based on prejudicial and intolerant views about different social groups (Hawdon et al., 2015 in Blaya et al., 2020; Wachs et al., 2019, 2020). Perpetrators of bullying may hold prejudicial views, but that is not always the case. In contrast, prejudice was said to always be present in cyberhate, even if it was directed towards an individual rather than a group (Wachs et al., 2019). With regards to targets, Räsänen et al. (2016) differentiates online hate and cyberbullying by arguing that hate material aimed to denigrate groups (in the sense of a collective to which individuals belong). Instead, cyberbullying is framed as an attack on individuals. Lehmann (2019, p. 537) spoke of “larger categories […] who are a general target of hate.” Blaya et al. (2020, p.2) referred to “the identity and community to which individuals belong.” The description of hate speech or cyberhate as an open signal of inter-group hostility (Hawdon et al., 2015 in Blaya & Audrin, 2019; Lehmann, 2019) points to the derogatory content of the expression. Looking at the effects of hate speech and bullying, the publications analyzed suggest that hate speech was potentially more harmful to human relationships than bullying in that it stigmatized marginalized out groups (Lehmann, 2019) and weakened social cohesion, democracy, and human rights (Blaya & Audrin, 2019).

Another criterion to differentiate hate speech from verbal bullying was the sequencing of both phenomena. Online bullying was contrasted with online hate in that the former was seen as a repeated activity whereas the latter could manifest itself as a single act (Wachs et al., 2019a, 2019), occur occasionally, or be triggered by external events (Blaya et al., 2020). The studies suggested that the similarities and differences between hate speech and bullying were associated with perpetrator- and target-related characteristics, as well as the forms, modes, sequencing, and effects of both expressions.

### Empirical Overlap of Hate Speech and Related Phenomena

Findings on empirical associations between bullying and online hate speech were reported in four publications (Blaya et al., 2020; Blaya & Audrin, 2019; Lehmann, 2019; Wachs et al., 2019). With reference to Cohen (1988), a number of studies identified correlations ranging from minor, for cyberbullying perpetration and cyberhate victimization (*r* = .15, *p* < .001, Wachs et al., 2019), to quite substantial, for cyberhate victimization and traditional bullying victimization at school (*r* = .47, *p* < .001, Blaya et al., 2020). The correlation of cyberhate perpetration with traditional bullying perpetration at school was moderate (*r* = .30, *p* < .001, Blaya et al., 2020) and just marginally higher with cyberbullying perpetration (*r* = .32; *p* < 0.001; Wachs et al., 2019). Cyberbullying perpetration predicted cyberhate perpetration significantly and to a minor extent (*β* = .19, *p* < .001, Wachs et al., 2019. Further, cyberhate perpetration did overlap to a small extent with cyberbullying victimization (*r* = .19, *p* < .001, Wachs et al., 2019) and with traditional bullying victimization (*r* = .19, *p* < .001; Blaya et al., 2020). Wachs et al., 2019 reported that cyberbullying victimization predicted cyberhate perpetration only to a very small extent (*ß* = .06, *p* = .013). For traditional bullying victimization, Blaya & Audrin (2019) found that, from their survey of cyberhate perpetrators (N = 100), a total of 51.4% (n = 73) were never bullied (insulted, threatened, or excluded) during the 6 months prior to the survey. But 43.6% (n = 61) indicated that they had experienced some form of bullying at least once and 11.3% (n = 11) stated that that they had been bullied five times or more.

No publications or studies assessed the empirical overlap between bullying and offline hate speech. Lehmann (2019) only tested whether the explanatory models for hate speech and bullying victimization were statistically similar. He applied the method of seemingly unrelated regression analysis (Felmlee & Hargens, 1988) and found that the presence of school security and/or police only predicts increased odds of students reporting hate-related (but not bullying-related) outcomes. Lehmann (2019) concluded that the statistical modeling of victimization through bullying is not identical to victimization based on the use of hate-related words, but it would be “premature to say that hate and bullying are empirically distinct” (Lehmann, 2019, p. 548). In their respective theory sections, Räsänen et al. (2016) and van Dorn (2004) did not refer to overlapping concepts or phenomena, but they reported relevant empirical results. Räsänen et al. (2016) found that the likelihood of online hate victimization was 3.6 times higher (*OR* = 3.57, *95% CI* = 2.39, 5.34, *p* < .001) if respondents reported offline victimization (being attacked or threatened in the past 3 years by a known person and in a way that they considered particularly scarring) compared to respondents who did not report such an experience. In the school context, van Dorn (2004) stated that students who reported that they were violently victimized in school (e.g., via attempted or perpetrated robbery, rape, or assault with a weapon) were three times more likely than their non-victimized counterparts to report hate-related words or phrases being used against them (*OR* = 2.97, *95% CI* = 1.72, 5.15, *p* < .001). Being non-violently victimized (e.g., via attempted or perpetrated theft) did not significantly increase the odds of reporting hate-related victimization (*OR* = 1.46, *95% CI* = .97, 2.21, *p* = .07).

In summary, cyberhate and bullying (both on- and offline) did appear to partly overlap in adolescent samples in Germany, France, and the US. For the sample of US-based adolescents, the empirical modeling of bullying and hate speech differed. It was further shown that, for samples in France, Finland, and the US, victimization experiences of different kinds (e.g., being bullied in person or in cyberspace; being attacked offline) co-occurred with cyberhate victimization or cyberhate perpetration. Appendix 3 reports empirical evidence that has been identified on the overlap of hate speech and other related phenomena. Table 3 summarizes the key findings of this systematic review.

**Table 3.** Critical Findings of this Systematic Review.

Definitions of hate speech and differentiation from other phenomena
• A majority of definitions referred to online hate speech • Medium-scope definitions were more common than broad or narrow definitions • Common frameworks for hate speech were behavior/action, aggression/violence, and communication/expression • Hate speech was most frequently differentiated from bullying
Prevalence of hate speech and empirical overlap with other phenomena
• Most studies used self-assessments with single or multiple items • Very few studies assessed all participating roles (exposure, perpetration, victimization) • Samples’ age varied from 12 to 21 years • The majority of publications were at least of medium quality • Exposure (26% to 69%) was more common than victimization (7% to 24%) or perpetration (5% to 32%) • Correlations across roles of hate speech and bullying were mostly small in size

## Discussion

Hate speech is a prevalent topic in public discourse but a systematization of academic literature on prevalence rates among children and adolescents was lacking. In this systematic review, we identified 18 publications, based on 10 studies, which contained empirical data on the prevalence of hate speech among children and young people. All of these studies focused solely on adolescents and not on younger children and the majority of publications appeared within the last 6 years, indicating a rise in research on young people and hate speech.

### Definitions of Hate Speech and Theoretical Differentiation from Bullying

The comparison of hate speech definitions across publications revealed that there is currently no standard or consensus-based definition of hate speech. In a few publications, no definitions were given at all (e.g., Elpus & Carter, 2016). Medium-scope definitions were used more frequently than broad or narrow definitions. The scope of the definitions even varied across publications taken from the same studies (e.g., Wachs & Wright, 2018, 2020). This reflects researchers’ ongoing attempts to refine the definition of hate speech. It is common in research on adolescents to frame hate speech as an expression or more generally as an action or behavior, to state the common targets of hate speech (e.g., groups and/or individuals), to name group categories (e.g., religion, sexual orientation etc.), and to consider the consequences of hate speech, generally for individuals (e.g., feeling humiliated), but sometimes for communities too (e.g., reduction of social cohesion). Four publications (Oksanen et al., 2014; Räsänen et al., 2016; van Dorn, 2004; Wachs et al., 2019) explicitly cited intentions, attitudes, or emotions in their descriptive features of hate speech. Implicitly, an intention to harm can be attributed to definitions that classify hate speech as a form of aggression or violence (Lehmann, 2019; Wachs et al., 2019; Wachs & Wright, 2018, 2019), because such an intention is a defining feature of these general terms.

The authors of those publications that were systematized generally theorized similarities and differences between hate speech and bullying. A partial overlap with regard to forms, modes, sequencing, and perpetrator characteristics was acknowledged in theoretical terms. No criteria appeared in any of the texts that precisely differentiated between the concept and phenomenon of hate speech or bullying. The demarcation was only given based on a joint consideration of various criteria. We suggest that hate speech should be distinguished from non-physical forms of bullying like insults. A specific difference between hate speech and bullying is the content of the related verbal expressions. Insults denigrate the characteristics of specific individuals, whereas hate speech denigrates the characteristics of specific social groups. This suggested differentiation implies that, throughout on- and offline contexts, single or repeated incidents of hate speech may be experienced alongside repeated incidents of bullying, but that hate speech can also occur independently from bullying. Future qualitative research should aim to explore the microdynamics between perpetrators, victims, and bystanders in events in which both bullying and hate speech are present. There might be a difference, for instance, between an occasion in which a perpetrator of bullying used hate speech, insults, or threats with an identical victim and another situation in which someone perpetrates hate speech anonymously on social media, but does not use it when bullying a classmate. Recent interdisciplinary debates on hate speech definitions have also dealt with related terms, such as harassment or discrimination (for Germany see Sponholz, 2020; Wachs et al., 2021b). The linguistic properties of hate speech, compared to other forms of derogatory expressions, such as ethnophaulisms, slurs or epithets, do overlap (Cervone et al., 2021) and also require more theoretical clarification. Going forward, researchers should contribute to the interdisciplinary debate on hate speech at both a national and international level, and they should aim to critically reflect on any definitional consensus achieved in their publications.

The features of hate speech that this review has identified are similar to those found by Titley et al. (2014, p.9), who reported that most of the hate speech definitions they reviewed referred to content, intentions, targets, and consequences. But, in that review, the assessed definitions covered these features inconsistently. Based on our review, we would offer the following definition of hate speech:

Hate speech is a derogatory expression (e.g., words, posts, text messages, images, videos) about people (directly or vicariously) on the basis of assigned group characteristics (e.g., ethnicity, nationality, gender, sexual orientation, disability, religion). Hate speech is based on an intention to harm and it has the potential to cause harm on multiple different levels (e.g., individual, communal, societal).

This proposed definition has four main features. The general category “derogatory expression” (feature one) is a combined term and specifies human behavior. It also captures digital forms of expression (e.g., pictures and videos) and it covers on- and offline contexts alike. Forms and modes can also be deduced from the general term “derogatory expression.” The suggested definition, in full, illustrates forms with examples that are taken from both on- and offline contexts, thus not requiring modes and forms as separate definitional features. It is recommended that researchers studying the phenomenon in its online modus refer to online hate speech, but may also choose to introduce cyber hate and online hate as synonyms. This will simplify the tracking and integration of academic research that covers derogatory expressions about people on the basis of assigned group characteristics across on- and offline modes. A target relation (feature two) is apparent in the content of the expression as referring to “people (directly or vicariously) on the basis of assigned group characteristics.” The definition purposely avoids limiting itself to the denigration of currently marginalized, structurally oppressed, or minority groups, because it is important to acknowledge that other social categories may become a target of hate speech in the future. Though it is debatable as to whether a public context is an essential feature of hate speech (for an overview, see Sponholz, 2020) our proposed definition omits this term. This decision was taken for two reasons. Firstly, there is a substantial amount of overlap in the concepts of public and private in the digital age (a “context collapse,” Sellars, 2016, p. 29). This limits one’s ability to precisely define any given phenomenon related to these realms. Secondly, hate speech may also be experienced in institutions (e.g., private or public schools) for which legal definitions of the public sphere may not be appropriate. The term “derogatory expression” is also structured using a consequence relation (feature three), which is explained as potentially causing “harm on different levels.” Biased attitudes (e.g., prejudice) or emotions (e.g., hate, contempt) have been omitted in the proposed definition in favor of an “intention to harm” (feature four). By considering intent, hate speech is conceptually linked with verbal violence or verbal aggression.

Although four publications defined hate speech based on attitudes or emotions, and only two explicitly referred to violence or aggression (see results section), it can be argued, using the theory of reasoned action (Fishbein & Ajzen, 2010), that intentions are proximal to human behavior. Being aware that a speaker’s intent may be obscured, denied, not disclosed, that speakers may lie about their intent, and that online identification is difficult (Sellars, 2016), the definition implies that the consideration of intent is crucial to balancing out principles of speech. If intent were to be disregarded, then hate speech would become a catch-all term for a broad range of offensive language and the term would lose its potential to identify those derogatory expressions which are seriously harmful to the peaceful coexistence of people in a pluralistic society and which thus require the concerted effort of multiple actors in society, including in academia.

The term “harm” encompasses negative physical and non-physical consequences (e.g., promotion of violence, making a person feel demeaned, vilified or humiliated, silencing members of an out group). We use the term “potentially” to make clear that the definition is sensitive to the fact that “it is not always obvious what speech does or does not incite and how it is altered, recontextualized and appropriated by the audience” (Sellars, 2016, p. 28). By referring to different levels of harm, the definition locates hate speech, its social functions (for an overview, see Cervone et al., 2021), and its consequences in a socio-ecological context that is sensitive to hate speech exposure, victimization, and perpetration alike. On the one hand, the suggested definition attempts to match the complexity of the phenomenon of hate speech while differentiating it in terms of related phenomena. On the other, the definition aims to serve as a conceptual reference point for operationalizing the concept of hate speech in support of empirical research.

### Prevalence of Hate Speech and Empirical Overlap with Related Behaviors

The analysis of publications on the prevalence of hate speech amongst young people has revealed a variety of definitions and assessment characteristics and an uneven availability of data on hate speech in terms of exposure, victimization, and perpetration. The results of this review indicate that, to date, no psychometric instrument has yet been established that allows us to generate valid, reliable, and comparable data across all types of participation in hate speech. Future research should aim to create just such an instrument, one that is linked to a concise definition, capturing all relevant features. The complexity of hate speech demands an assessment based on multiple-item scales, rather than using single items and global evaluations. The methodological debate on the assessment of bullying and cyberbullying provides useful reference points in this area (for an overview, see Menesini & Nocentini, 2009). The definition of hate speech proposed above supports indicator-guided methods of hate speech detection (e.g., as recommended by Sellars, 2016 and recently piloted by Papcunova et al., 2021) and it may be applied in connection with survey-based, observational, and automated methods to assess the prevalence of hate speech.

Social contexts which are structured by historical, political, cultural, and legal characteristics encompass specific community relations. They also entail descriptive and prescriptive speech norms, laws and speech practices (e.g., Bilewicz & Soral, 2020). Future studies and publications should specify the social context in which they aim to locate hate speech. They should come up with a shared set of factors that guide a comparative perspective on quantitative and qualitative research alike. A standardized psychometric assessment should also take into account the distinction of hate speech and related behaviors, for instance bullying.

The empirical results on prevalence rates, group differences, overlap with related behaviors, and control variables (such as age, gender, socio-demographic factors etc.) are based on only a few studies and publications. More evidence might increase their validity. The results on hate speech exposure ranged from a minimum of 26% (12–18-year-olds in the US; see Lehmann, 2019, 2020) to a maximum of 68% (12–18-year-olds in Spain; see Wachs et al., 2019). None of the reviewed publications targeted children younger than 12-years old. The sparsity of research on hate speech frequencies related to younger children is echoed by a general lack of studies on Internet usage in this group (Holloway et al., 2013). However, children start to use the Internet at an early age (Holloway et al., 2013) and six-to-nine-year-olds are, at times, exposed to frightening or violent content online (Chaudron et al., 2018). Thus, the question of what age children are first exposed to hate speech should also be addressed in future research.

Hate speech and bullying co-occur in the experience of perpetrators and victims. The results of this review revealed small to moderate associations between cyberhate and school bullying (Blaya & Audrin, 2019; Blaya et al., 2020) and small associations between cyberhate and cyberbullying (Wachs et al., 2019). More broadly, offline victimization also overlaps with both on- and offline hate speech (Räsänen et al., 2016; van Dorn, 2004). For this reason, research on children and adolescents and efforts to prevent or intervene in hate speech and bullying must take the wider context of both behaviors into account. Besides the family, schools and teachers play a vital role in democracy and media education (Wachs et al., 2021b). A socio-ecological framework (Bronfenbrenner, 1994), a mixed method design (Schoonenboom & Johnson, 2017), and multi-level analytics (e.g., Snijders & Bosker, 2011) thus all represent useful tools to guide both research and practical efforts alike.

### Limitations and Implications of this Review

Overall, the findings of this study contribute to our understanding of research on hate speech among young people. However, some methodological limitations must be acknowledged. The search-strategy may have missed relevant publications, because the review only searched for publications in English and German, meaning that relevant findings published in other languages were not identified. Future systematic reviews might also include a more extensive network-approach (e.g., via mailing lists) in order to identify preprint material or studies that have not been listed in the databases accessed. A quality assessment of the whole study—on which several publications are based—might also be a subject of interest for future reviews. Table 4 summarizes the implications of this systematic review for practice, policy and research.

**Table 4.** Implications for Practice, Policy, and Research.

Implications for practice	The findings of this review serve practitioners by enabling them: (1) to efficiently access the academic research that deals with the prevalence of hate speech and overlap issues focused on children and adolescents, and (2) to base prevention and intervention efforts (e.g., educational programs) on an academic knowledge base
Implications for policy	The study is useful for policy-makers by enabling them: (1) to root policy (e.g., laws, guidelines) on a critically reflected broad definition of hate speech, and (2) to use country-specific data regarding the prevalence of hate speech and its overlap with bullying to align their policies with empirical evidence
Implications for research	This review identified areas that should be considered in future research. It is recommended that researchers: (1) participate in cross-disciplinary (non-) academic debates on hate speech and strive for definitional consensus, (2) develop and use standardized tools to assess the prevalence of hate speech among children and adolescents in order to gain valid, reliable and comparable results, (3) conduct more empirical research on factors associated with an overlap between hate speech and other phenomena, and (4) conduct studies targeted at younger children (below age 12)

## Conclusion

The present study fills existing gaps in the research literature on hate speech by systematizing prevalence rates on hate speech among children and adolescents (5 to 21 years) and on hate speech definitions that guided the prevalence assessment for this population; and by systematizing research on the theoretical and empirical overlap of hate speech and related concepts. It was found that definitions of hate speech and assessment instruments were considerably heterogeneous and that more adolescents were exposed to hate speech rather than experiencing it as a victim or perpetrator. The review also revealed that, although hate speech and bullying theoretically share a number of features, they only partially overlap empirically. A precise definition of such a complex and widely debated phenomenon as hate speech requires a critical evaluation of meaning and usage in public and academic discourse. The proposed definition of hate speech that we have offered here supports current academic research on hate speech as it relates to children and adolescents. As such, this review facilitates a structured access to research on hate speech among young people.

## Appendix 1

### Coding Features

**Table:**

Basic information about the publication	Country of data-collection Type of publication (e.g., journal, PhD etc.) Year of publication Research approach (quantitative/qualitative/mixed methods)
Theoretical foundation	Definition of hate speech (yes/only contextual/no; if yes: term/text & author*) Definition of related concepts (yes/no; if yes: term/text & author*) Inherent features of the definition (e.g., sensitive to modus, groups, forms, locations etc.)* Theoretical assumptions on overlap (yes/no; if yes: text*)
	Theory applied within study*
Information on methodology	Sample age Sample size Sample context (e.g., elementary or secondary school, internet) Sampling strategy (e.g., random, lump-selection) Fit between definition of hate speech and prevalence measure Empirical analysis of conceptual overlap (yes/no) *Characteristics of assessing hate speech prevalence* Specific item characteristics (e.g., sensitive to modi & forms) *Assessment of overlap* Empirical assessment of overlap (yes/no)
	Concept for which overlap was assessed*
Results	Topic (e.g., hate speech prevalence in relation to health, media literacy, psychological consequences, interventions)* General research question(s)* Research question(s) that address prevalence of hate speech* Hypotheses regarding overlap (yes/no; if yes: text*) General results Prevalence of hate speech (perpetration/exposure/victimization)* Empirical overlap between hate speech and other phenomenon*

## Appendix 2

### Terms and Scope of Hate Speech Definitions.

**Table:**

Publication	Applied Term	Derived General Terms	Derived Descriptive Terms (DT)^1^	N_DT_	Scope^2^
Blaya & Audrin (2019)	Cyberhate	Communication, expression	Forms, targets, effects, perpetrator, context	5	Medium
Blaya et al. (2020)	Cyberhate	Expression	Forms, targets, effects, perpetrator, publicity, context	6	Narrow
Lehmann (2019)	Hateful written words & graffiti, hate	Group dynamics	Targets, effects	2	Broad
Lehmann (2020)	Hateful written words & graffiti, hate	Incivility, group dynamics	Targets, effects	2	Broad
Oksanen et al. (2014)	Hate material	Act, expression	Forms, targets, effects, perpetrator, context	5	Medium
Räsänen et al. (2016)	Hate material, hate speech, online hate	Activity, communication	Targets, effects, perpetrator, context	4	Medium
van Dorn (2004)	Hate speech, hate-related words	Symbols	Forms, targets	2	Broad
Vaught (2012)	Racist hate speech	Mechanisms, messages	Forms, targets, effects, perpetrator	4	Medium
Wachs & Wright (2018)	Online hate	Action	Targets, effects, context	3	Broad
Wachs & Wright (2019)	Online hate	Aggression, behavior	Targets, perpetrator, context	3	Broad
Wachs & Wright (2020)	Online hate	Action	Forms, targets, effects, perpetrator, context	5	Medium
Wachs et al. (2019)	Cyberhate	Aggression	Targets, effects, context	3	Broad
Wachs et al. (2019)	Online hate	Aggression, expression	Forms, targets, directness, perpetrator, context	5	Medium
Wachs et al. (2020)	Cyberhate	Action	Forms, targets, effects, perpetrator, context	5	Medium
Wachs et al. (2021a)	Cyberhate, hate speech	Expression	Forms, targets, directness, effects, perpetrator, context	6	Narrow

^1^Forms: e.g., derogatory words, pictures, videos, graffiti. Targets: e.g. marginalized groups, in-/out-groups, individuals. Effects: e.g. harm or benefits for individuals, in-/out-groups, society. Perpetrator: e.g., aims/purpose/intent to harm, hate, prejudice, toxic online disinhibition. Context: e.g., online/offline modus; locations. Publicity: publicly, privately. Directness: absence/presence of targeted individual or group.

^2^Scope based on the total number of descriptive terms: broad: 1–3, medium 4–5, narrow: >6.

## Appendix 3

### Overlap between Hate Speech and other Related Phenomena (Correlations, Standardized Regression Weights, Odds Ratios, 95% Confidence Intervals).

**Table:**

Type of Overlap	Coefficient (*r, ß, OR*), 95% CI [Lower, upper]
*Cyberhate and traditional bullying*	Blaya et al. (2020)
Cyberhate victimization and traditional bullying victimization	*r* = .47 [.391, .547]
Cyberhate victimization and traditional bullying perpetration	*r* = .19 [.085, .299]
Cyberhate perpetration and traditional bullying victimization	*r* = .19 [.086, .283]
Cyberhate perpetration and traditional bullying perpetration	*r* =.30 [.144, .447]

*Cyberhate and cyberbullying*	Wachs et al. (2019a)
Cyberhate victimization and cyberbullying victimization	*r* = .28
Cyberhate victimization and cyberbullying perpetration	*r* =.15
Cyberhate perpetration and cyberbullying victimization	*r* =.19; *ß* = .06^1^ [0.014, 0.118]^3^
Cyberhate perpetration and cyberbullying perpetration	*r* =.32; *ß* = .19^2^ [0.129, 0.241]^3^

*Hate speech and offline victimization*	
Cyberhate victimization and offline victimization	Räsänen et al. (2016) *OR* = 3.577 [2.393, 5.346]
Offline hate victimization and other offline victimization	van Dorn (2004) *OR*_violently_ = 2.97 [1.72, 5.15] *OR*_nonviolently_ = 1.46 [.97, 2.21]*

*Note.* All measures (despite *) are highly significant (p =/< .001). Regression weights were controlled for grade, gender, socio-economic status, migration background.

^1^Prediction of cyberhate perpetration by cyberbullying victimization.

^2^Prediction of cyberhate perpetration by cyberbullying perpetration.

^3^Confidence intervals are related to regression weights.
